# Salivary profile in diabetic patients: biochemical and immunological evaluation

**DOI:** 10.1186/s13104-016-1881-1

**Published:** 2016-02-16

**Authors:** Monica Virginia Viegas Lima-Aragão, João de Jesus de Oliveira-Junior, Márcia Cristina Gonçalves Maciel, Lucilene Amorim Silva, Flávia Raquel Fernandes do Nascimento, Rosane Nassar Meireles Guerra

**Affiliations:** Laboratory of Immunophysiology, Campus Universitário do Bacanga, Universidade Federal do Maranhão (UFMA), 1966-Centro, São Luís, MA 65080 Brazil

**Keywords:** Saliva, Composition, Diabetes mellitus, IgA, Glucose, Urea, Calcium

## Abstract

**Background:**

The aim of this study was to evaluate the biochemical and immunological characteristics of saliva from diabetic patients compared to non-diabetic adults.

**Methods:**

Eighty-eight diabetic adults and 39 non-diabetic adults (control) were included in the study. Glucose, urea, calcium, total protein and amylase were determined by a colorimetric method. The levels of secretory IgA and the IgA anti-*Streptococcus mutans* and anti-insulin IgA antibodies were measured by enzyme-linked immunosorbent assay (ELISA). Caries status was evaluated using the DMFT index.

**Results:**

Glucose, urea, calcium, anti-*S. mutans* IgA, total IgA, and anti-insulin IgA were significantly higher in diabetic patients, whereas total protein and amylase levels were lower in these patients. There was no positive correlation between blood and salivary glucose levels in either group. Diabetic patients had a higher DMFT index.

**Conclusions:**

The present study showed for the first time that IgA levels in diabetic patients’saliva, shows correlation with systemic biochemical parameters. Thus the saliva is an useful tool to follow the systemic health status in these patients.

## Background

Diabetes mellitus is a public health problem since, in addition to important social repercussions. It is a chronic disease that affects a growing number of individuals from different countries which are at different stages of economic and social development [1]. Diabetes is classified into four clinical types: type 1 diabetes which results from β-cell destruction and usually leads to absolute insulin deficiency; type 2 diabetes which results from a progressive insulin secretory defect associated with insulin resistance; other specific types of diabetes due to other causes, and gestational diabetes mellitus [2].

Systemic diseases such as diabetes compromise salivary gland function and consequently influence the quantity and quality of saliva produced. Alterations in the chemical components and physical properties of saliva can serve as diagnostic parameters and saliva testing may therefore be added to the arsenal of complementary tests [3]. Saliva has several advantages over serum, including its simple and noninvasive collection and easy storage, transport and handling [4]. The wide variety of components found in saliva permits its use for diagnosis, prognosis and monitoring of human diseases [5], such as hereditary or congenital diseases [6], autoimmune diseases, cardiovascular diseases, infections [7], cancer [8], diabetes [9], caries [10], and periodontal disease [11], among others.

Differences in saliva production and composition have been observed previously between diabetic and non-diabetic subjects [12, 13]. Some authors have shown variable correlation rates between serological and saliva data [9, 12, 14]. However, despite this apparent controversial the saliva seems to be important and useful to evaluate physiologic alterations.

The aim of the present study was to evaluate the biochemical and immunological profile of saliva in diabetic patients, considering the total production of IgA, and the concentration specific IgA anti-insulin and anti-*Streptococcus mutans*, as additional biomarkers in saliva to state the differences between diabetic and non diabetics patients.

## Methods

### Population

The sample size was calculated based on the prevalence of diabetes in Maranhão (MS Hiperdia [15]), with a confidence interval of 95 %. Data were collected systematically during 6 month. The sample consisted of 142 subjects (93 diabetics and 49 controls).

The population was divided into two groups age-sex matched: DIABETIC and NON DIABETIC. The diabetic group comprises patients with diabetes type I and Type II, since there are no statistic differences among them, with a previous diagnosis of more than 4 years.

This study used a convenience sample based on the following inclusion criteria: age older than 18 years, a previous diagnosis of diabetes mellitus, and participation in the Diabetes Program of Presidente Dutra University Hospital, São Luís, Maranhão, Brazil. Only 88 diabetic patients, who met the inclusion criteria, had sufficient salivary flow for analysis and effectively agreed to participate in the study. The following criteria were used for inclusion in the control group: age older than 18 years, no systemic disease or diabetes, and no history of medication use. Only 39 non-diabetic adults, who met the inclusion criteria, effectively participated in the study. Sociodemographic data were obtained by semi-structured interview.

All subjects received information regarding the objective and procedures of the study and only participated in the data collection after signing a free informed consent form. The study protocol was approved by the Ethics Committee of Universidade Federal do Maranhão (Protocol No. 33104-149).

### Collection of saliva samples

Unstimulated whole saliva was collected, in fasting, from diabetic and non-diabetic subjects over a period of 5 min. After rinsing their mouth with filtered water, the subjects were asked to spit saliva into a sterile flask. The flasks were sealed immediately after sample collection and refrigerated at 4 °C before transport to the laboratory. The saliva samples were centrifuged (10,000 rpm, 10 min) to reduce salivary debris and viscosity. Glucose and total protein concentrations were determined immediately after centrifugation. The other parameters were evaluated later in a supernatant stored at −20 °C.

### Biochemical analysis

Glucose, total protein, urea, calcium and amylase concentrations were determined by a colorimetric method. The analyses were performed using automated procedures on an Architect C8000 apparatus (Abbott^®^) for glucose, amylase, total protein and calcium. Individual samples were tested in triplicate.

### Determination of IgA levels

Salivary total IgA, anti-*S. mutans* IgA and anti-insulin IgA antibodies were determined by enzyme-linked immunosorbent assay (ELISA) as described previously [11]. Briefly, anti-human IgA (Sigma, St. Louis, USA) was diluted to a concentration of 50 µg/mL in carbonate-bicarbonate buffer (pH 9.6) and adsorbed onto ELISA strips in flat-bottom wells (NUNC, Roskilde, Denmark), followed by overnight incubation at 4 °C. The wells were then washed five times with 0.15 M phosphate-buffered saline (PBS; pH 7.2) containing 0.05 % Tween 20 (Promega, Madison, USA; PBS-T) and incubated in PBS containing 1 % bovine serum albumin (Sigma) for 1 h at 37 °C. The wells were washed five times and 100 µL of tenfold dilutions (1:100 to 1:1000) of saliva samples in PBS was added. After 30 min at 37 °C and further washing, 100 µL alkaline phosphatase-labeled goat anti-human IgA (Sigma; diluted 1:1000 in PBS-T) was added to the wells and the strips were incubated for 2 h at 37 °C. The strips were then washed five times with PBS-T. The color was developed by adding 100 µL of a solution of ρ-nitrophenylphosphate (Sigma) and incubating the strips for 30 min in the dark. The reaction was stopped with 1 N NaOH (Sigma) and the optical density was measured at a wavelength of 405 nm.

### Clinical examination

The caries status of the patients was evaluated using the DMFT (decayed, missing and filled permanent teeth) index and was performed by a single examiner. Data on oral hygiene, access to dental care, and salivary flow were also collected.

### Statistical analysis

Quantitative variables were compared between the two groups by the Student *t* test. The correlation between diabetic and non-diabetic patients was evaluated using Pearson’s linear correlation coefficient. A receiver operating characteristic (ROC) curve was constructed for the diagnostic test. This analysis considered glucose, total protein, urea, anti-insulin Ig, and amylase concentrations. The test was defined as positive when alterations in at least four parameters were observed. The sensitivity of the test was 88 %, specificity was 90 %, and diagnostic accuracy was 89 %. Categorical variables were compared by Fisher’s exact test. The level of significance for rejection of the null hypothesis was set at p ≤ 0.05 in all tests. All analyses were performed using the GraphPad Prism 5.0 program.

## Results

The control group consisted of 39 clinically healthy subjects with a mean (±SD) age of 23 ± 6 years (range: 19–50 years). The mean (±SD) age of the diabetic patients was 52 ± 18 years (range: 18–84 years).

Fifty-seven of the 88 diabetic patients were females and 31 were males, and 59 were using insulin. The time since diagnosis of diabetes was 11 ± 8 years. Blood glucose levels were 261 ± 131 mg/dL. The data obtained for diabetic patients were compared to a control (non-diabetic) group of 39 subjects, including 22 females and 17 males. The mean (±SD) blood glucose levels were 92 ± 9 mg/dL in this group.

**Fig. 1:**
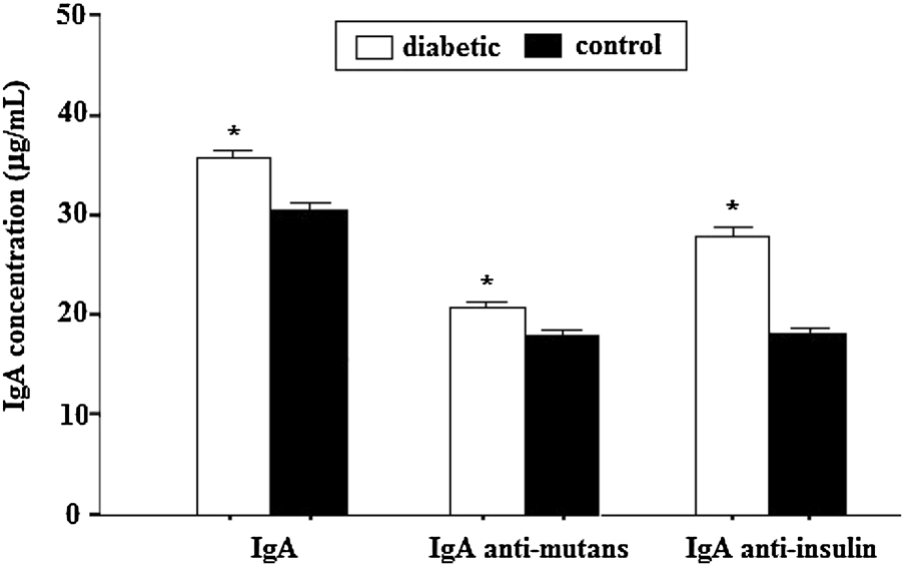
Salivary concentration of total IgA, anti-mutans (*Streptococcus mutans*) IgA and anti-insulin IgA in diabetic patients and healthy controls. The results are reported as the mean ± standard deviation (X ± SD) of 88 diabetic patients and 39 controls. (*) Significant when p < 0.05 compared to the control group

Table 1 shows the salivary biochemical parameters and Fig. 1 illustrates the immunological parameters. Glucose, urea, calcium, anti-*S.mutans* IgA, total IgA and anti-insulin IgA were significantly higher in diabetic patients (p < 0.05). On the other hand, total protein and amylase concentrations were significantly lower in diabetic patients (p < 0.05).

**Table 1:** Salivary parameters of diabetic and non-diabetic patients

Parameter	Control^a^	Diabetica	p value
Glucose (mg/dL)	3 ± 0.03^b^	11 ± 2	0.01
Urea (mg/dL)	17 ± 1	27 ± 1	0.0001
Calcium (mg/dL)	2 ± 0.1	2 ± 0.1	0.0004
Total protein (mg/dL)	2 ± 0.1	0.3 ± 0.03	0.0001
Amylase (AU/dL)	37 ± 0.4	37 ± 0.1	0.008

^a^Saliva samples obtained from 39 non-diabetic (control) subjects and 88 diabetic patients of both genders were evaluated

^b^Results are reported as the mean ± standard deviation (X ± SD)

No positive correlation was observed between blood and salivary glucose levels in either group, although glucose levels were higher in diabetic patients than in the control group (Table 1). A negative correlation was found between salivary glucose concentration and anti-insulin IgA.

The diabetic patients were divided into two subgroups according to the use or not of insulin. The results showed no significant differences between diabetes I or diabetes II patients. For this reason, the salivary data were evaluated together as diabetic patients (Table 2).

**Table 2:** Salivary composition of diabetic patients who used insulin or not

Parameter	Use of insulin		p value
	Yes	No	
Glucose	12 ± 3^b^	9 ± 3^b^	0.51
Urea	28 ± 1	25 ± 1	0.06
Calcium	2 ± 0.1	2 ± 0.2	0.41
Amylase	0.3 ± 0.1	0.2 ± 0.1	0.56
Total protein	37 ± 1	37 ± 1	0.57
Anti-mutans IgA	21 ± 1	20 ± 2	0.59
Total IgA	35 ± 1	36 ± 1	0.61
Anti-insulin IgA	28 ± 2	27 ± 2	0.51

^a^Saliva samples obtained from 88 diabetic patients of both genders were evaluated

^b^Results are reported as the mean ± standard deviation (X ± SD)

A ROC curve was constructed to validate the salivary parameters that could be used for diagnostic testing. Glucose, total protein, urea, anti-insulin IgA, amylase and calcium concentration were included in the analysis. Only calcium was excluded after ROC analysis since it exhibited an area of 0.5, i.e., this parameter was not statistically significant (Table 3).

**Table 3:** Area under the ROC curve obtained for salivary parameters of diabetic patients

Parametera	Area under the ROC curve	Standard error	p value
Glucose	0.99	0.01	<0.0001
Total protein	0.98	0.02	<0.0001
Amylase	0.95	0.02	<0.0001
Anti-insulin IgA	0.84	0.04	<0.0001
Urea	0.81	0.05	<0.0001
Calcium	0.55	0.06	0.4274

^a^Saliva samples obtained from 88 diabetic patients of both genders were evaluated

Clinical examination showed a high DMFT index in diabetic patients (Table 4). Furthermore, compared to the control group, these patients had poor oral hygiene and less access to dental care and exhibited xerostomia signs such dry mouth (Table 5).

**Table 4:** Index of decayed, missing and filled permanent teeth (DMFT) in diabetic patients

Index	Diabetic (n = 88)	Control (n = 39)	p value
D	4 ± 0.4^a^	1 ± 0.2	<0.001
M	16 ± 1	0.5 ± 0.2	<0.001
F	1 ± 0.2	3 ± 0.4	<0.001
DMFT^b^	20 ± 1	5 ± 0.5	<0.001

^a^Results are reported as the mean ± standard error of the mean

^b^ *DMFT* decayed, missing, filled permanent teeth

**Table 5:** Oral hygiene, access to dental care and dry mouth sensation in diabetic patients and controls

	Diabetic f (%)^a^	Control f (%)^a^	p value^b^
Number of tooth brushing			
1–2	52	10	<0.0001
3–4	48	90	
Total	100	100	
Use of dental floss			
Yes	22	67	<0.0001
No	78	33	
Total	100	100	
Last dental visit			
1 year	23	61	<0.0001
2–3 years	77	38	
Total	100	100	
Dry mouth sensation			
Yes	63	5	<0.0001
No	37	95	
Total	100	100	

^a^f (%), frequency expressed as percentage

^b^Fisher’s exact test

## Discussion

Whole saliva is a mixture of salivary gland secretions containing substances derived from gingival crevicular fluid, desquamated epithelial cells, food rests, microorganisms, and products derived from microbial metabolism [16]. Since stimulation affects both the production of saliva and the concentration of some of its components [3, 17], in the present study unstimulated whole saliva was used to evaluate oral alterations associated with diabetes in adult patients.

A significant increase in the salivary concentration of glucose, calcium and urea was observed in diabetic patients. Similar results of calcium concentration have been reported previously [18]. Furthermore, the increases were similar to those reported by other investigators that evaluated biochemical parameters in the saliva of diabetic patients [12]. Changes in salivary composition have been suggested to affect the development, symptoms and severity of many oral diseases, particularly in diabetic patients [9].

No correlation between salivary and blood glucose levels was observed in diabetic patients despite higher salivary glucose concentration in this group. Salivary and blood levels of glucose may be considered independent variables as previously described for adults [12–14] and children with diabetes [19]. According to Jurysta et al. [13], salivary glucose concentration measured either in unstimulated or stimulated saliva is always higher in diabetic patients than in control subjects, and this high concentration exerts potentially unfavorable effects on the oral health of these patients. These results agree with the present findings and confirm the weak association between blood glucose levels and glucose concentration or excretion in saliva, at least in individual cases [14].

Total amylase and protein were significantly lower in diabetic patients. Amylase was evaluated as a marker of metabolic and hormonal changes in salivary gland products. Yavuzyilmaz et al. [20] also reported lower levels of amylase in diabetic patients. In contrast, salivary total protein concentration differed from that reported in studies evaluating this parameter after freezing and storage of the sample [21]. This difference might be explained by the fact that in the present study the assay was performed immediately after collection and centrifugation of the saliva samples to prevent endogenous proteolytic activity [4].

Evaluation of immunological changes in saliva showed a higher total IgA antibody concentration in diabetic patients. These findings are probably associated with the elevated levels of specific anti-*S. mutans* and anti-insulin antibody levels also observed in these patients. The determination of changes in whole saliva composition may be used to diagnose possible variations and defective host immune responses, including the secretion of s-IgA [22]. Similar results have been reported in other studies evaluating the salivary production of IgA antibodies in diabetic patients [20, 23].

According to Batista et al. [23], *S. mutans* counts are usually higher in diabetic patients. Although the degree of infection of the patients was not quantified in this study, elevated anti-*S. mutans* antibody titers might be related to an increased bioavailabilityof bacterial antigens. The local response of specific antibodies to certain plaque bacteria such as *S. mutans* may identify sites with a potential risk of developing caries and other oral diseases even before onset of the first clinical signs [24]. *Streptococcusmutans* is a Gram-positive bacterium that is potentially aggressive to various tissues. Elevated anti-*S. mutans* IgA concentrations may indicate that the patients are systematically exposed to this microorganism, as well as a higher caries risk [25], especially if we consider the poor oral hygiene and less access to dental care showed in the present study (Table 5).

In the present study high levels of anti-insulin IgA antibodies were detected in saliva from diabetic patients and this result may explain the elevation of glucose levels observed in diabetic patients. The diabetes hyperglycemia Insulin is known to have a regulatory role on glucose levels and the anti-insulin antibodies are frequently responsible to block the insulin ligation to the respective receptor, increasing the biodisponibility of this hormone and decreasing its action [26–28], resulting from defects in insulin secretion, insulin action, or both [2], observed in diabetes.

There is no previous report about anti-insulin antibodies in saliva from diabetic patients in association with other salivary profile. For this reason the results altogether may highlight information about the salivary biomarkers in diabetic patients, since the detection of anti-insulin antibodies are frequently associated to insulin antibody-induced insulin resistance and can be used as diagnostic and prognostic markers to diabetes, but also to other autoimmune diseases [26, 27].

According Eisenbarth (2003) [28], specific patterns of autoantibody expression may predict risk and progression to diabetes ranging from 20 % to >90 %, mainly in diabetes type I. We propose a detection of IgA anti-insulin in saliva as useful tool to predict diabetes risk early, as well as, an important result to monitoring the progression of disease in diabetic patients, with the same accuracy as that obtained for serum anti-insulin antibodies and with less discomfort to the patients during fluid collection.

Considering a large number of patients were using injectable insulin, we investigated whether this treatment alters the variables analyzed. No significant differences in salivary biochemical or immunological parameters were observed between non-insulin-dependent and insulin-dependent diabetic patients. Similar findings have been reported by Jurysta et al. [13] and Yavuzyilmaz et al. [20] who also compared saliva of insulin-dependent and non-insulin-dependent diabetic patients.

Biochemical and immunological alterations in the saliva of diabetic patients have been described and salivary changes may serve as a complementary parameter for the diagnosis of diabetes mellitus [3]. In the present study, the clinical performance of an individual test was demonstrated by the ROC curve after the variables had been combined and were validated for diagnostic use. The greater the capacity of a test to discriminate between diabetic and non-diabetic subjects, the closer is the curve to the left corner of the graph, with an area under the curve close to 1 [29]. The sequence of individual variables that best discriminate diabetic patients was glucose (area of 0.99), total protein (area of 0.98), amylase (area of 0.95), anti-insulin IgA (area of 0.83), and urea (area of 0.80). The accuracy of the diagnostic test combining these parameters was 89 %. A similar accuracy (90 %) has been reported by Lopez et al. [19] who used saliva for the diagnosis of diabetes in children considering calcium, urea, total sugars, glucose and total protein concentration as done in the present study for adult patients.

The association of dental caries with diabetes is less clear. In this study, diabetic patients had a higher DMFT index than control subjects. The average number of teeth lost was 16 and the average number of decayed teeth was 3.7, which were responsible for the high DMFT index in these patients. The dry mouth sensation and increased salivary glucose levels observed here may alter the effectiveness of salivary protection against caries [10]. Other factors such as poor oral hygiene and deficient regular care of these patients can aggravate pre-existing diseases. Data from clinical evaluation may explaining the high level of IgA *anti*-*S.mutans,* as low salivary flow, and high DMFT index are frequently associated to an increase in bacterial counts and the risk of infection among diabetic patients.

Its important to emphasizes the age differences between the control group (23 ± 6, range: 19–50 years) and diabetic patients (52 ± 18), as well the range intra group (range: 18–84 years), due to the inclusion criteria What can also influence the differences found between the two groups.

Diabetic patients with reduced salivary flow have an increased risk of enamel hypomineralization and caries formation [30]. A decrease in salivary flow can cause oral alterations such as an increase in the concentration of mucin and glucose. Proliferation of pathogenic microorganisms may also occur. The consequences of chronic hyperglycemia include tongue alterations, periodontal disease, tooth demineralization, and an increased infection risk [31]. The early detection and treatment of hyperglycemia and hyposalivation may provide a useful strategy to prevent the dental complications of diabetes and to promote oral health in this population [32].

Salivary composition in diabetes type 1 and 2 can be correlated to an increased risk of periodontal disease [33, 34]. Carda et al. [16], evaluating type 2 diabetic patients, found symptoms of periodontal disease associated with tooth decay and tooth loss in all patients. Jones et al. [35] demonstrated an increased caries risk in 457 patients with diabetes mellitus. Their study included patients with type 1 and type 2 diabetes, similar to the present study.

There was a positive correlation between DMFT and total IgA and a negative correlation between DMFT and tooth brushing in diabetic patients. Similar results have been reported by Smith and Taubman [36] for adult patients. In the group with caries, the authors observed a significant positive correlation between plaque index and specific IgA.

The diagnostic value of saliva has been demonstrated in a series of studies showing the accuracy and specificity of this body fluid for the diagnosis of diseases [3–5, 7–9, 12, 13, 17, 19, 20, 37]. The ease of collection of saliva samples and the lower risk of contamination of healthcare workers during sample handling are important advantages that support the use of this fluid as a diagnostic tool. Furthermore, studies comparing the results of saliva analysis with other reliable scientific methods, such as blood measurements, have demonstrated that the method is reliable and its application is therefore valid.

There is no previous report about the detection of anti-insulin in saliva of diabetic patients and its relation to several biochemical parameters. The present study was the first to demonstrate that a combination of immunological and biochemical parameters as useful data to monitor diabetic patients, since significant differences were observed in the saliva composition of diabetic patients, The alterations detected indicating a strong relationship between our findings and the systemic health status of the patient. Moreover, some salivary parameters were found to be useful to classify an adult as diabetic. This difference in salivary composition thus suggests the use of saliva as an alternative fluid to monitor patients with diabetes mellitus, considering the increase of diabetes not only in Brazil but also in other countries and the possibility to find new forms of diagnosis to design prevention strategies.

## Abbreviations

*IgA*: immunoglobulin A
s-IgA: secretory IgA
ELISA: enzyme-linked immunosorbent assay
DMFT index: decayed, missing and filled permanent teeth index
ROC curve: receiver operating characteristic
X ± SD: mean ± standard deviation

## References

[1] Wild S, Roglick G, Green A, Sicree R, King H (2004). Global prevalence of diabetics: estimates for the year 2000 and projections for 2030. Diabetes Care.

[2] American Diabetes Association (2012). Standards of medical care in diabetes. Diabetes Care.

[3] Moura SAB, Medeiros AMC, Costa FRH, Moraes PH, Oliveira filho SA (2007). Valor diagnóstico da saliva em doenças orais e sistêmicas: uma revisão da literatura. Pesq Bras Odontop Clin Integr..

[4] Bigler LR, Streckfus CF, Dubinsky WP (2009). Salivary biomarkers for detection of malignant tumors that are remote from oral cavity. Clin Lab Med.

[5] Oliveira Júnior JJ (2010). Guerra RNM. Biomarcadores imunológicos da saliva. RevCiencSaude (São Luís).

[6] Grody WW (1999). Cystic fibrosis: molecular diagnosis, population screening, and public policy. Arch Pathol Lab Med.

[7] Pink R, Simek J, Vondrakova J, Faber E, Michl P, Pazdera J (2009). Saliva as a diagnostic medium. Biomed Pap Med FacUnivPalacky Olomouc Czech Repub.

[8] Guerra RNM, Oliveira-Junior JJ, Mouchrek-Filho JCE, Liberio AS, Lima MVV, Paim DBS, Brito CXL, Mendonça C, Nascimento FRF, Pereira ALA (2012). Salivary evaluation of pediatric patients with cancer, before and after antineoplastic treatment. J Oral Pathol Med.

[9] Negrato CA, Tarzia O (2010). Buccal alterations in diabetes mellitus. Diabetol Metabolic Syndr.

[10] Miko S, Smbrus SJ, Sahafian S (2010). Dental caries and adolescents with type 1 diabetes. Br Dent J.

[11] Branco-de-Almeida LS, Alves CMC, Lopes FF, Pereira AFV, Guerra RNM, Pereira ALA (2011). Salivary IgA and periodontal treatment needs in diabetic patients. Braz Oral Res..

[12] Carda C, Mosquera-Lloreda N, Salom L (2006). Gomez De Ferraris ME, Peydró A. Structuralandfunctionalsalivarydisorders in type 2 diabeticpatients. Med Oral Pathol Oral. Cir Bucal.

[13] Jurysta C, Bulur N, Oguzhan B, Satman I, Yilmaz TM, Malaisse WJ, Sener A (2009). Salivary glucose concentration and excretion in normal and diabetic subjects. J Biomed Biotech.

[14] Soares MSM, Batista-Filho MMV, Pimentel MJ, Passos IA, Küstner EC (2009). Determination of salivary glucose in healthy adults.Med Oral Pathol Oral. Cir Bucal.

[15] MS—Ministério da Saúde do Brasil-HIPERDIA: capturedon August 2011-site: http://hiperdia.datasus.gov.br/.

[16] Kaufman E, Lamster IB (2002). The diagnostic application of saliva. A review. Crit Rev Oral Biol.

[17] Mandel ID (1990). The diagnostic uses of saliva. J Oral Pathol Med.

[18] Monteiro AMD, Araújo RPC, Gomes Filho IS (2002). Diabetes Mellitus tipo 2 e Doença Periodontal. RGO.

[19] Lopez ME, Colloca ME, Páez RG, Schallmach JN, Koss MA, Chervonagura A (2003). Salivary characteristics of diabetic children. Braz Dent J.

[20] Yavuzyilmaz E, Yumar O, Akdoganli T, Yamalik N, Ozer N, Ersoy F, Yenia YI (1996). The alterations of whole saliva constituents in patients with diabetes mellitus. Aust Dent J.

[21] Flegal KM, Ezzati TM, Harris MI, Haynes SA, Juarez RZ, Knowler WC (1991). Prevalence of diabetes in Mexican Americans, Cubans, and Puerto Ricans from the Hispanic health and nutrition survey, 1982–1984. Diabetes Care.

[22] Bachrach G, Muster Z, Raz I, Chaushu G, Stabholz A, Nussbaum G (2008). Assessing the levels of immunoglobulins in the saliva of diabetic individuals with periodontitis using checkerboard immunodetection. Oral Dis.

[23] Batista JE, Batista CEM, MonteiroNeto V, Guerra RNM (2004). Colonização por *Streptococcusmutans* e dosagens de anticorpos antiinsulina na saliva de diabéticos. Rev CiêncSaúde (São Luís)..

[24] Bratthall D (1997). Immunoglobulin A reaction to oral streptococci in saliva of subjects with different combinations of caries and levels of mutans streptococci. Oral Microbiol Immunol.

[25] Koga-Ito CY, Martins CAP, Balducci I, Jorge AOC (2004). Correlation among mutans streptococci counts, dental caries, and IgA to *Streptococcus mutans* in saliva. Braz Oral Res.

[26] Yu L, Robles DT, Abiru N, Kaur P, Rewers M, Kelemen K, Eisenbarth GS (2000). Early expression of anti-insulin auto-antibodies of humans and the NOD mouse: evidence early determination of subsequent diabetes. Proc Natl Acad Sci.

[27] Kamradt T, Mitchison NA (2001). Advances in immunology: tolerance and autoimmunity. New Engl J Med.

[28] Eisenbarth G (2003). Insulin autoimmunity: imumunogenetics/immunopathogenesis of type 1A diabetes. Ann NY Acad Sci..

[29] Martinez EZ, Louzada-Neto F, Pereira BB (2003). A curva de ROC para testes diagnósticos. Cadernos de Saúde Coletiva.

[30] Moore PA, Guggenheimer J, Etzel KR (2001). Type 1 diabetes mellitus, xerostomia, and salivary flow rates. Oral Surg Oral Med Oral Pathol Oral RadiolEndod.

[31] Vernillo AT (2003). Dental considerations for the treatment of patients with diabetes mellitus. J Am Dent Assoc.

[32] Yeh CK, Harris SE, Mohan S, Horn D, Fajardo M, Chun YHP, Jorgensen J, MacDougall M, Abboud-Wener S (2012). Hyperglycemia and xerostomia are key determinants of tooth decay in type 1 diabetic mice. Lab Invest.

[33] Ben-Aryeh H, Serouya R, Kanter Y, Szargel R, Laufer D (1993). Oral health and salivary composition in diabetic patients. J Diabetes Complications.

[34] Arrieta J, Bartolomé B, Jiménez E, Saavedra P, Arrieta F (2003). Problemas bu-codentalesen pacientes con diabetes mellitus. Med Oral.

[35] Jones RB, McCallum RM, Kay EJ, Kirkin V, McDonald E (1992). Oral health and oral health behaviour in a population of diabetic clinic attenders. Community Dent Oral Epidemiol.

[36] Smith DJ, Taubman MA (1992). Ontogeny of immunity to oral microbiota in humans. Crit Rev Oral Biol Med.

[37] Lawrence HP (2002). Salivary markers of systemic disease: noninvasive diagnostic of disease and monitoring of general health. J Can Dent Assoc.

